# The Impact of Technology-Enabled Medical Nutrition Therapy on Weight Loss in Adults With Overweight and Obesity: Retrospective Observational Study

**DOI:** 10.2196/70228

**Published:** 2025-05-06

**Authors:** Emily A Hu, Tommy Kelley, Ajay Haryani

**Affiliations:** 1 Nourish New York, NY United States; 2 Department of Medicine Icahn School of Medicine at Mount Sinai New York, NY United States

**Keywords:** medical nutrition therapy, mHealth, obesity, overweight, registered dietitian, smartphone application, telehealth, virtual, weight loss

## Abstract

**Background:**

Obesity represents a major public health crisis in the United States, imposing substantial health risks and economic costs. Medical nutrition therapy (MNT) is an evidence-based treatment where a registered dietitian provides personalized nutrition and lifestyle guidance to patients. MNT has been demonstrated to be effective for weight loss and managing chronic diseases in patients with obesity. With the rise of telehealth, MNT has gained popularity as an accessible alternative to traditional in-person care. While a nationwide program integrating MNT with a companion mobile app offers a comprehensive weight management solution, data supporting its clinical effectiveness is limited.

**Objective:**

This study aimed to evaluate the effectiveness of an MNT program with a companion mobile app on weight loss among adults with overweight and obesity.

**Methods:**

This retrospective cohort study included users of Nourish, an MNT program with a companion mobile app, who attended at least 1 appointment between August 2023 and October 2024 and had a baseline BMI≥30 kg/m² or a BMI between 27-30 kg/m² with diabetes or prediabetes. The primary outcome was the proportion of participants who achieved at least 5% weight loss; secondary outcomes included mean weight change, mean percent weight change, and the proportion of participants who achieved at least 3% weight loss. Statistical significance of weight change was determined using 2-tailed *t* tests. Subgroup analyses were performed by sex, BMI, follow-up time between weights, number of appointments completed, and levels of engagement according to appointment frequency and app usage.

**Results:**

In total, 3951 participants were included in the analysis. The mean age was 38 (SD 10) years, and 78% (3082/3951) of participants were female. Weight loss was reported as a program goal by 70% (2748/3951) of participants, while 31% (1204/3951) and 24% (939/3951) reported diabetes or prediabetes and a cardiovascular condition, respectively. Over a median follow-up of 2.2 months, 17% (689/3951) of participants achieved at least 5% weight loss. The mean weight change was –4.5 (SD 8.9) pounds, corresponding to a mean percent weight change of –2% (SD 3.9; *P*<.001). Males and participants aged 60 years or older were more likely to experience at least 5% weight loss. Longer follow-up time between weights and a higher number of completed appointments (≥5 appointments) were significantly associated with a significantly higher likelihood of achieving at least 5% weight loss (*P*<.001 for both). In addition, participants who were most engaged, based on appointment frequency and app usage, were more likely to achieve at least 5% weight loss compared with those who were less engaged (*P*<.001).

**Conclusions:**

Engagement with an MNT program and companion mobile app is associated with significant weight loss for adults with overweight and obesity and may serve as an effective, scalable weight management solution.

## Introduction

Obesity remains a critical public health challenge nationwide, affecting over 100 million adults across the United States according to recent estimates [1]. In the United States, the prevalence of obesity has continued to rise, with over 40% of adults classified as obese in 2023 [2] and rates expected to exceed 60% in 2050, posing a significant public health threat given its impact on the existing chronic disease epidemic [3]. This trend is associated with substantial health and economic burden, as obesity-related conditions, such as diabetes and other cardiovascular disease risk factors, are projected to worsen cardiovascular health and cost up to US $1.49 trillion annually, along with additional losses from reduced productivity [4].

A cornerstone of obesity prevention and treatment is an optimization of behavioral and lifestyle modifications, including adherence to regular physical activity and a well-balanced, heart-healthy diet [5]. Effective nutritional counseling, as part of a medical nutrition program, has repeatedly proven to result in weight loss, thereby lowering an individual’s risk of obesity-related comorbidities, including diabetes and cardiovascular disease [6-10].

Medical nutrition therapy (MNT) is a treatment modality where a registered dietitian (RD) meets one-on-one with a patient to provide individualized lifestyle recommendations. MNT can be used to address a wide range of health conditions and may occur in various practice settings, such as outpatient and inpatient facilities. Following an initial intake, an RD meets with a patient regularly to individualize their treatment plan and help patients achieve their health goals. Data have consistently demonstrated the impact of MNT on reducing body weight, BMI, and waist circumference and have been shown to improve weight-related risk factors, including hypertension, diabetes, hyperlipidemia, and quality of life [6,8]. For weight management specifically, previous research has demonstrated that participants receiving MNT were twice as likely to achieve 5% weight loss compared with controls [6]. Despite its effectiveness, the utilization of MNT in practice is low relative to the prevalence of obesity for multiple reasons, including access, affordability, and awareness of this resource [11,12].

In recent years, mobile health interventions targeting weight management have gained popularity. These apps often feature calorie and meal logging, meal planning, and grocery ordering. Studies show that such apps can lead to significant short-term and long-term weight reductions [13-15]. However, many mobile apps lack tailored feedback, which may limit engagement and long-term use, underscoring the need for complementary interventions.

During the COVID-19 pandemic, the adoption of telehealth significantly broadened health care accessibility [16]. In response, the American Heart Association (AHA) issued a policy statement emphasizing the importance of expanding access to and optimizing the use of MNT, citing its numerous benefits for managing chronic diseases [17]. MNT delivered via telehealth, which emerged as an opportunity to bridge the gap highlighted by the AHA, had the potential to not only improve access and convenience of MNT but to also help achieve clinical goals. Emerging research has validated the potential of MNT delivered via telehealth to deliver on the AHA mission by improving access, and in fact, has resulted in better engagement and adherence resulting from the increased convenience of telehealth [18]. The emergence of telehealth is especially important for rural populations, who face unique challenges, such as limited access to health care providers and greater distances to fresh food sources [19,20]. These barriers are associated with worse health outcomes, particularly for those with chronic conditions. Telehealth offers a promising solution to address these disparities by providing on-demand access to health care services.

Starting in 2021, a national program, that combines MNT delivered via telehealth with a companion mobile app, was launched to provide care for individuals with overweight and obesity seeking to lose weight and improve their health. The app includes features such as meal logging, meal planning, and outcomes tracking. By combining 2 proven interventions for weight loss, the program sought to deepen patient engagement with their MNT treatment, and in turn, see meaningful clinical outcomes. This study aimed to evaluate the effectiveness of this combined solution on weight loss among adults with overweight and obesity across the United States.

## Methods

### MNT Program With a Companion Mobile App

Nourish is a national telehealth provider of MNT delivered by a licensed RD, paired with a companion mobile app to offer asynchronous support and tools. Nourish has a network of over 1500 RDs covering all major specialties, including overweight and obesity, diabetes, and heart disease, and can serve patients across all 50 states. Patients find Nourish independently or through a referral from a clinician. The cost of Nourish is covered by insurance for the majority of patients as Nourish is in a network with the nation’s largest commercial insurance plans. Upon enrollment, patients provide demographic information, medical history, program goals, and personal preferences, which are used to match patients with recommended RDs that best fit their clinical needs and goals. Patients then schedule their first appointment with an RD they would like to work with. Patients have the option to switch providers at any time during their program.

**Figure 1:**
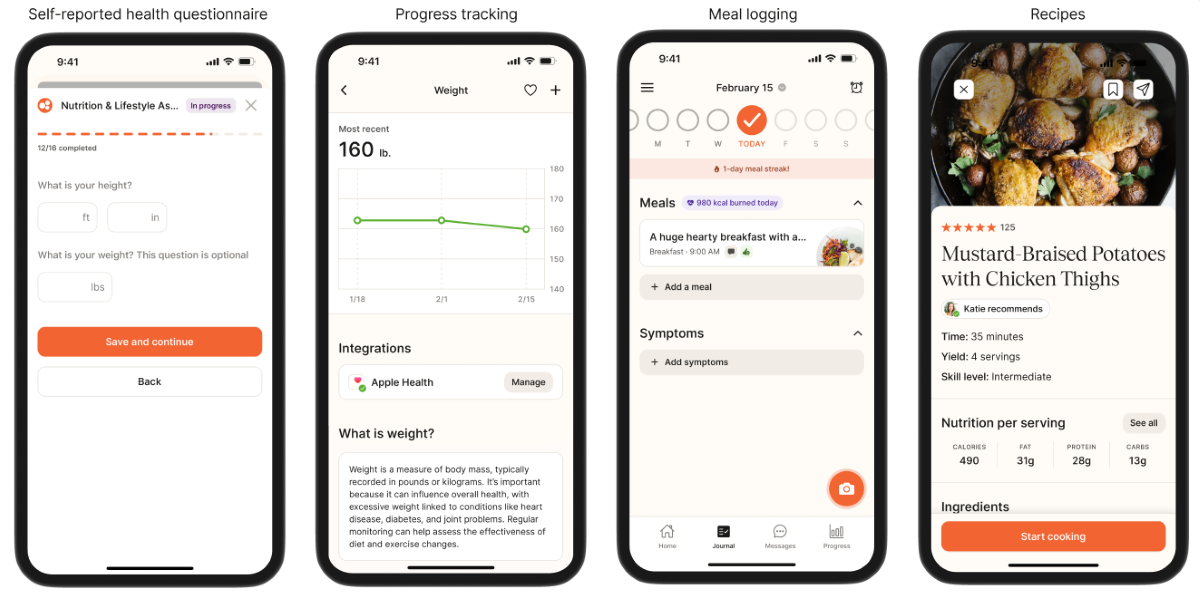
Screenshots of Nourish mobile app.

Patients meet virtually in one-on-one sessions with their RD for an initial nutritional intake assessment through the Nourish web portal or app. The RD then develops a personalized treatment plan focused on nutrition and lifestyle recommendations to help patients achieve their health goals. Furthermore, 1-hour sessions are scheduled every 1 to 3 weeks, depending on the treatment plan. In addition to telehealth appointments, patients also have access to a proprietary mobile app (Android [Google], iOS [Apple Inc]) and web portal with features designed to complement the clinical intervention and increase engagement. Features of the app include a self-reported health questionnaire that assesses lifestyle and nutritional habits, progress tracking of weight and other physiological measurements and laboratory data, meal logging, recipe and meal planning content, nutrition education resources, and on-demand chat with an RD (Figure 1). Patients receive automated reminders to track their progress and log meals through push notifications within the app and can also set up customized reminders. Patients also receive notifications to schedule their next appointment.

### Study Population

This retrospective cohort study evaluated weight change and engagement among newly enrolled users who completed their first appointment with an RD between August 2023 and October 2024. Individuals were eligible for inclusion if they were at least 18 years old and met one of the following BMI criteria: baseline BMI≥30 kg/m^2^ or baseline BMI between 27 and 30 kg/m^2^ with self-reported diabetes or prediabetes. In addition, we required individuals to have at least 2 nonidentical weight measurements at least 30 days apart with their first weight within 2 weeks of their first appointment and their last at least 4 weeks after their first appointment and within 90 days of their last appointment. Exclusion criteria included reported usage of weight loss medications, history of bariatric surgery, or history of thyroid disorders. Given the retrospective and observational nature of this study, a prospective sample size and power analysis were not performed.

### Assessments

Data used for this analysis were self-reported by participants in the app or during the appointment, where RDs entered data on the participants’ behalf. In addition, platform-collected app engagement data were used to derive the number of appointments, app touchpoints, and time between recorded weights. Participants were prompted to enter demographic and clinical information when they enrolled in the program. These self-reported assessments included sex, age, weight, height, and goals for the program (eg, weight loss, managing comorbidities such as diabetes or prediabetes, and cardiovascular conditions such as high blood pressure and high cholesterol). Participants could update their weight at any time through the app, and RDs were prompted to record weight during appointments if participants did not update them in the app. All fields were optional except for date of birth and goal for enrolling in the program.

Based on self-reported height and weight data, BMI was calculated. Weight change was calculated as the difference between baseline weight and follow-up weight. Percent weight change was calculated as weight change divided by baseline weight. Follow-up time between weights was defined as days between the date that baseline weight was recorded and the date that follow-up weight was recorded. In addition, follow-up time between appointments for participants who attended at least 2 appointments was calculated. The primary outcome was the proportion of participants who achieved at least 5% weight loss. Secondary outcomes included mean weight change (in pounds), mean percent weight change, and the proportion of participants who achieved at least 3% weight loss. Additional outcomes included the proportion of participants who achieved any weight loss, at least 7% weight loss, and at least 10% weight loss.

To examine the relationship between engagement and weight reduction, engagement metrics based on app-generated data were also analyzed. Engagement was assessed based on the number of completed appointments and the number of total touchpoints with the app. The number of touchpoints with the app was calculated based on total engagements with key app features, including the number of meals logged, the number of messages sent to their RD, and the number of vital or laboratory values (eg, weight, height, blood pressure, and cholesterol) entered.

### Statistical Analyses

Baseline characteristics were summarized for the total population and further stratified by whether participants achieved at least 5% weight loss. Descriptive statistics, including means, SDs, medians, IQRs, and ranges for continuous variables and percentages for categorical variables were reported. Group comparisons were conducted using ANOVA for continuous variables and chi-square tests for categorical variables. Furthermore, 2-tailed *t* tests were conducted to determine whether weight changes significantly differed from zero. Participants with missing data for age and sex were excluded from the analyses for those specific metrics.

Mean (SD) weight change, mean (SD) percent weight change, and the proportion of participants who achieved at least 5% weight loss were calculated by baseline characteristics. The mean (SD) weight change and mean (SD) percent weight change were calculated for the total population and stratified by whether participants achieved at least 5% weight loss, with results presented by sex and BMI category. ANOVA and chi-square tests were used to test for significant differences within each subgroup.

To address potential bias from loss to follow-up, the proportion of participants achieving at least 3% and 5% weight loss were analyzed according to follow-up time between weights (<2 months, 2 to up to 3 months, 3 to up to 6 months, and ≥6 months) and the number of appointments completed (1-2, 3-4, 5-7, and ≥8 appointments). Furthermore, the proportion of participants who achieved any and at least 3%, 5%, 7%, and 10% weight loss was calculated among the total population and by whether participants completed at least 5 appointments (the mean number of appointments in the study population). To evaluate the potential interaction of appointments and app touchpoints on weight loss, participants were stratified into low- or high-engagement groups using mean values as cutoffs to compare relative engagement within the study population. In total, four subgroups were defined: (1) low appointments (<5) and low app usage (<100 touchpoints), 2) low appointments (<5) and high app usage (≥100 touchpoints), (3) high appointments (≥5) and low app usage (<100 touchpoints), and (4) high appointments (≥5) and high app usage (≥100 touchpoints).

A *P* value less than .05 was considered to be statistically significant for all tests. Stata (version 18; StataCorp) was used for all analyses.

### Ethical Considerations

This study adhered to ethical standards for human participants research. At sign-up, all participants were notified that their data may be used for research purposes, and data were deidentified before analysis to ensure privacy. No compensation was provided to participants. The study was declared exempt from institutional review board oversight by Advarra institutional review board (protocol #00083062) given the retrospective design of the study and less than minimal risk to participants.

## Results

### Participant Characteristics

In total, 3951 participants were included in this analysis. Baseline characteristics are presented in Table 1. Participants had a mean age of 38 (SD 10) years, mean BMI of 37 (SD 6) kg/m^2^, and were primarily female (3082/3951, 78%). Furthermore, 52% (2066/3951) of participants had a BMI greater than 35 kg/m^2^ and a majority (2748/3951, 70%) reported that weight loss was a goal of the program. Diabetes or prediabetes and cardiovascular conditions were reported among 31% (1204/3951) and 24% (939/3951) of participants, respectively. The mean and median follow-up time between weight entries was 80 (SD 49) days and 67 (IQR 47-95) days, respectively. The mean and median time between appointments was 70 (SD 61) days and 52 (IQR 26-95) days. In total, participants attended a total of 18,966 appointments (mean 4.8, SD 4.0) and had 393,131 touchpoints with the app (mean 100, SD 101; data not shown).

**Table 1:** Baseline characteristics.

Characteristics		Total population (N=3951)	Achieved ≥5% weight loss (n=689)	Achieved <5% weight loss (n=3262)	*P* value
**Sex^a^, n (%)**					
	Male	759 (19)	151 (22)	608 (19)	.05
	Female	3082 (78)	514 (75)	2568 (79)	—^b^
**Age** ^a^ **(y)**					
	Mean (SD)	38 (10)	38 (11)	38 (10)	.02
	Median (IQR)	36 (30-44)	36 (31-44)	36 (30-43)	—
	Range	18-78	19-78	18-78	—
**Age group (y), n (%)**					
	18-29	809 (20)	138 (20)	671 (21)	.03
	30-39	1653 (42)	280 (41)	1373 (42)	—
	40-59	1237 (31)	209 (30)	1028 (32)	—
	≥60	141 (4)	38 (6)	103 (3)	—
**Follow-up time between weights (d)**					
	Mean (SD)	80 (49)	97 (53)	76 (47)	<.001
	Median (IQR)	67 (47-95)	85 (62-116)	63 (45-90)	—
	Range	30-424	30-403	30-424	—
**Follow-up time between appointments^c^ (d)**					
	Mean (SD)	70 (61)	81 (67)	67 (60)	.002
	Median (IQR)	52 (26-95)	64 (29-112)	49 (24-91)	—
	Range	1-412	3-361	1-412	—
**Weight (pounds)**					
	Mean (SD)	228 (44)	229 (44)	227 (44)	.4
	Median (IQR)	220 (195-252)	220 (196-255)	220 (195-252)	—
	Range	131-398	142-396	131-398	—
**BMI (kg/m^2^)**					
	Mean (SD)	37 (6)	37 (6)	37 (6)	.4
	Median (IQR)	35 (32-40)	36 (32-40)	35 (32-40)	—
	Range	27-75	27-75	27-67	—
**Baseline BMI, n (%)**					
	27 to <30 kg/m^2^	181 (5)	28 (4)	153 (5)	.80
	30 to <35 kg/m^2^	1704 (43)	287 (42)	1417 (43)	—
	35 to <40 kg/m^2^	1048 (27)	193 (28)	855 (26)	—
	40 to <50 kg/m^2^	834 (21)	149 (22)	685 (21)	—
	≥50 kg/m^2^	184 (5)	32 (5)	152 (5)	—
	Diabetes or prediabetes, n (%)	1204 (31)	227 (33)	977 (30)	.10
	Cardiovascular condition, n (%)	939 (24)	168 (24)	771 (24)	.70
	Weight loss goal, n (%)	2748 (70)	487 (71)	2261 (69)	.50

^a^Overall, 3% (n=151) of the total population did not report sex nor age and were not included in summaries of sex and age.

^b^Not applicable.

^c^Among participants who had at least 2 appointments (n=3269).

### Weight Change

In total, 17% (689/3951) of participants achieved at least 5% weight loss (Table 2). When comparing results by sex, males were more likely to achieve at least 5% weight loss compared with females (151/759, 20% vs 514/3082, 17%; *P*=.05, 95% CI 1.1-4.9). Participants who were 60 years or older were more likely to achieve at least 5% weight loss compared with participants who were younger (38/141, 27% vs 627/3699, 17%; *P*=.03, 95% CI 5.2-14.2). While those who had a higher baseline BMI lost more weight compared with those with a lower BMI (*P*<.001, 95% CI –6.0 to –3.0), there was no statistically significant association between BMI and achieving 5% weight loss. Notably, participants who entered the program with a goal of losing weight experienced significantly more weight loss than those without a weight loss goal (*P*<.001, 95% CI –1.8 to –0.6). However, the weight loss goal was not statistically and significantly associated with achieving at least 5% weight loss. In total, 74% (2929/3951) of participants lost any weight, 34% (1350/3951) of participants achieved at least 3% weight loss, 9% (336/3951) achieved at least 7% weight loss, and 3% (123/3951) achieved at least 10% weight loss (Table S1 in Multimedia Appendix 1).

**Table 2:** Proportion of participants who achieved at least 5% weight loss, mean weight change, and percent weight change by baseline characteristics

Population			Participants, N		Achieved ≥5% weight loss							Weight change (pounds)							Percent weight change (%)				
					n (%)		*P* value		95% CI		Mean (SD)			*P* value		95% CI		Mean (SD)			*P* value		95% CI
Total population			3951		689 (17)		—^a^		—		–4.5 (8.9)			—		—		–2 (3.9)			—		—
**Sex^b^**																							
	Male	759		151 (20)		.05		1.1 to 4.9		–6.1 (9.4)			<.001		–2.6 to –1.4		–2.4 (3.8)			.002		–0.9 to –0.3	
	Female	3082		514 (17)		—		—		–4.1 (8.7)			—		—		–1.8 (3.9)			—		—	
**Age group^b^ (y)**																							
	18-29	805		138 (17)		.03		5.2 to 14.2		–4.0 (9.2)			.20		–2.5 to –1.1		–1.7 (4.2)			.05		–1.5 to –0.5	
	30-39	1653		280 (17)		—		—		–4.6 (8.4)			—		—		–2 (3.7)			—		—	
	40-59	1240		209 (17)		—		—		–4.7 (9.3)			—		—		–2 (4)			—		—	
	≥60	142		38 (27)		—		—		–5.8 (7.9)			—		—		–2.7 (3.6)			—		—	
**Baseline BMI, %**																							
	27-<30 kg/m^2^	181		28 (16)		.80		0.5 to 4.3		–3.5 (7.8)			<.001		–6.0 to –3.0		–1.9 (4.5)			.02		–1.1 to –0.1	
	30-<35 kg/m^2^	1704		287 (17)		—		—		–3.5 (8.2)			—		—		–1.7 (4.1)			—		—	
	35-<40 kg/m^2^	1048		193 (18)		—		—		–4.7 (9)			—		—		–2 (3.9)			—		—	
	40-<50 kg/m^2^	834		149 (18)		—		—		–5.8 (9.6)			—		—		–2.2 (3.6)			—		—	
≥50 kg/m^2^			184		32 (17)		—		—		–8.0 (10.6)			—		—		–2.5 (3.2)			—		—
**Diabetes or prediabetes**																							
	Yes	1204		227 (19)		.10		0.9 to 3.2		–5.2 (9)			.002		–1.7 to –0.3		–2.2 (4.0)			.02		–0.6 to –0.1	
	No	2747		462 (17)		—		—		–4.2 (8.8)			—		—		–1.9 (3.9)			—		—	
**Cardiovascular condition**																							
	Yes	939		168 (18)		.70		–0.6 to 1.9		–5.0 (8.3)			.10		–1.2 to 0.0		–2.1 (3.6)			.10		–0.3 to 0.3	
	No	3012		521 (17)		—		—		–4.4 (9.1)			—		—		–2.1 (3.6)			—		—	
**Weight loss goal**																							
	Yes	2748		487 (18)		.50		–0.4 to 2.0		–4.9 (8.6)			<.001		–1.8 to –0.6		–2.1 (3.7)			<.001		–0.8 to –0.2	
	No	1203		202 (17)		—		—		–3.7 (9.6)			—		—		–1.6 (4.4)			—		—	

^a^Not applicable.

^b^Overall, 3% (n=151) of the total population did not report sex nor age and were not included in summaries for sex and age.

The mean weight change was –4.5 (SD 8.9) pounds (*P*<.001) and the mean percent weight change was –2% (SD 3.9; *P*<.001; Table 3). Among those who achieved at least 5% weight loss, the mean weight change was –17.6 (SD 6.3) pounds (*P*<.001) and the mean percent weight change was –7.7% (SD 2.4; *P*<.001), while those who did not achieve at least 5% weight loss had a mean weight change of –1.8 (SD 6.6) pounds and a mean percent weight change of –0.7% (SD 3.0; *P*<.001). While males lost more weight compared with females (mean weight change –6.1, SD 9.4 pounds for males vs mean weight change –4.1, SD 8.7 pounds for females), the mean percent weight change for those who achieved at least 5% weight loss was similar across males (mean –7.7%, SD 2.2) and females (mean –7.7%, SD 2.5). In addition, participants with a higher baseline BMI experienced greater weight loss compared with those with a lower BMI. Mean weight change was –3.4 (SD 8.2) pounds for participants with BMI<35 kg/m², –4.7 (SD 9.0) pounds for those with BMI 35-40 kg/m², and –6.2 (SD 9.8) pounds for those with BMI≥40 kg/m², demonstrating a progressive increase in weight loss with higher BMI categories.

**Table 3:** Mean weight change and percent weight change among the total population and stratified by 5% weight loss among all participants, by sex, and BMI category.

Category		Total population	*P* value^a^	Achieved ≥5% weight loss	*P* value^a^	Achieved <5% weight loss	*P* value^a^
**All participants**							
	n	3951	—^b^	689	—	3262	—
	Weight change (pounds), mean (SD)	–4.5 (8.9)	<.001	–17.6 (6.3)	<.001	–1.8 (6.6)	<.001
	Percent weight change (%), mean (SD)	–2.0 (3.9)	<.001	–7.7 (2.4)	<.001	–0.7 (3.0)	<.001
**Males**							
	n	759	—	151	—	608	—
	Weight change (pounds), mean (SD)	–6.1 (9.4)	<.001	–19.5 (6.0)	<.001	–2.7 (6.9)	<.001
	Percent weight change (%), mean (SD)	–2.4 (3.8)	<.001	–7.7 (2.2)	<.001	–1.1 (2.8)	<.001
**Females**							
	n	3082	—	514	—	2568	—
	Weight change (pounds), mean (SD)	–4.1 (8.7)	<.001	–17.0 (6.1)	<.001	–1.6 (6.6)	<.001
	Percent weight change (%), mean (SD)	–1.8 (3.9)	<.001	–7.7 (2.5)	<.001	–0.7 (3.0)	<.001
**BMI<30 kg/m^2^**							
	n	1885	—	315	—	1570	—
	Weight change (pounds), mean (SD)	–3.4 (8.2)	<.001	–15.6 (5.4)	<.001	–1.1 (6.3)	<.001
	Percent weight change (%), mean (SD)	–1.7 (4.4)	<.001	–7.9 (2.6)	<.001	–0.5 (3.2)	<.001
**BMI 30-35 kg/m^2^**							
	n	1048	—	193	—	855	—
	Weight change (pounds), mean (SD)	–4.7 (9.0)	<.001	–17.5 (5.3)	<.001	–1.8 (6.8)	<.001
	Percent weight change (%), mean (SD)	–2.0 (3.9)	<.001	–7.5 (2.2)	<.001	–0.8 (3.0)	<.001
**BMI** **≥35** **kg/m** ^**2**^							
	n	1018	—	181	—	837	—
	Weight change (pounds), mean (SD)	–6.2 (9.8)	<.001	–21.1 (7.0)	<.001	–3.0 (7.0)	<.001
	Percent weight change (%), mean (SD)	–2.2 (3.5)	<.001	–7.6 (2.3)	<.001	–1.1 (2.5)	<.001

^a^*t* test to test whether weight change was different from zero.

^b^Not applicable.

### Engagement With Program

**Figure 2:**
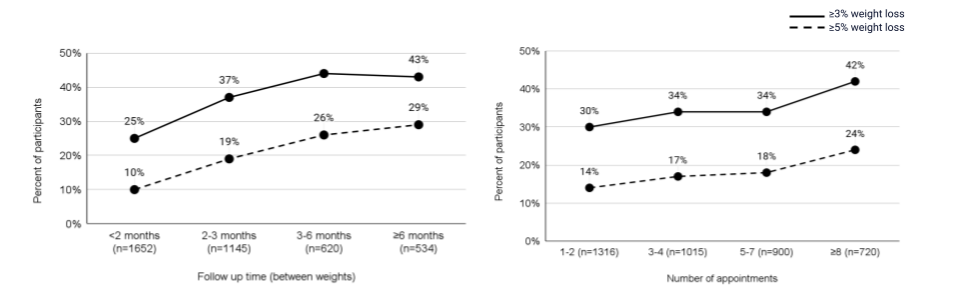
Percent of participants who achieved at least 3% and 5% weight loss according to (A) follow-up time and (B) number of appointments.

The proportion of participants who achieved at least 3% and 5% weight loss, stratified by time between weights, is presented in Figure 2A. Participants who engaged with the program for longer periods of time were more likely to achieve at least 3% (*P*<.001, 95% CI 15.0-20.8) and 5% weight loss (*P*<.001, 95% CI 16.2-21.9). Figure 2B illustrates the proportion of participants achieving at least 3% and 5% weight loss, stratified by the number of completed appointments (1-2, 3-4, 5-7, and ≥8). Participants who attended more appointments had a significantly greater likelihood of achieving at least 3% weight loss (*P*<.001, 95% CI 9.4-14.7) and 5% weight loss (*P*<.001, 95% CI 7.5-12.3).

Those who completed at least 5 appointments (n=1620) had significantly higher rates of achieving weight loss milestones compared with those who attended fewer than 5 appointments (n=2331): 38% (613/1620) vs 32% (737/2331) for at least 3% weight loss (*P*<.001, 95% CI 3.6-8.4), 20% (331/1620) vs 15% (358/2331) for at least 5% weight loss (*P*<.001, 95% CI 2.8-7.2), 11% (179/1620) vs 7% (157/2331) for at least 7% weight loss (*P*<.001, 95% CI 2.1-5.9), and 5% (73/1620) vs 2% (50/2331) for at least 10% weight loss (*P*<.001, 95% CI 1.4-4.6; Table S1 in Multimedia Appendix 1).

### Engagement With MNT and App

A significant interaction was observed between appointments and app engagement in predicting the likelihood of achieving at least 5% weight loss (*P*<.001, 95% CI 4.1-9.9; Table 4). Participants with high engagement in both appointments (≥5 appointments) and app usage (≥100 total touchpoints) had the greatest likelihood of achieving at least 5% weight loss. The likelihood of this occurrence followed in descending order by participants with a high number of appointments and low app usage, a low number of appointments and high app usage, and low appointment completion and low app usage.

**Table 4:** Percent of participants who achieved at least 5% weight loss by engagement data.

App usage^a^	Appointments^b^	
	Low (<5 appointments)	High (≥5 appointments)
Low (<100 touchpoints)	15% (280/1833)	19% (165/875)
High (≥100 touchpoints)	16% (78/498)	22% (167/745)

^a^App usage includes meal logging, messages to registered dietician, or health questionnaire entries.

^b^Percents (n/N) are displayed.

## Discussion

### Principal Findings

Among adults with overweight and obesity across the United States, enrollment in an MNT program with a companion mobile app was associated with clinically meaningful weight loss. Over a median of 67 (IQR 47-95) days, 17% (689/3951) of participants lost at least 5% of initial weight and the majority (2929/3951, 74%) of participants lost any weight. Males and participants older than 60 years old were more likely to achieve at least 5% weight loss compared with females and participants younger than 60 years old, respectively. The greatest weight loss was achieved by participants who were most engaged with both MNT appointments and the mobile app, suggesting a significant benefit of integrating both resources into a nutritional therapy solution.

MNT has been proven to be an effective strategy to treat obesity by achieving significant weight loss. A systematic review and meta-analysis of 14 randomized controlled trials comparing individualized nutrition care for weight management with usual care found a pooled mean difference of –2.3 pounds with 95% CI –3.1 to –1.5 (*P*<.001) over a median of 6 months [8]. In this study, participants who engaged with an MNT program experienced a mean weight change of –4.5 (SD 8.9) pounds over a median of 67 (IQR 47-95) days. Of note, several of the studies in the systematic review were focused on primary outcomes of blood pressure or diabetes, rather than weight loss. This suggests that when MNT is implemented with the primary goal of achieving weight loss, potentially more meaningful weight loss can be achieved.

In this study, while as few as 1 appointment resulted in meaningful weight loss, a higher number of appointments and longer time engaged in the program were directly correlated with a higher likelihood of clinically significant weight loss. In fact, the greatest outcomes were observed for participants who completed at least eight 1-hour appointments. Therefore, in addition to prioritizing clinically effective MNT, an effective program must also emphasize long-term engagement and adherence to optimize clinically meaningful weight loss for individuals. In fact, this was substantiated by the Academy of Nutrition and Dietetics who reported that the most beneficial outcomes for blood pressure and fasting glucose resulted after at least 5 sessions with an RD over a period of at least 12 months [21].

Although MNT helps individuals successfully lose weight, uptake has been low over the years, in part due to accessibility, affordability, and convenience [11,12]. At the same time, there has been a rise in digital health interventions for weight loss through mobile and web-based applications [22,23]. Several analyses evaluating the effectiveness of these commercial apps have been published and report meaningful weight loss among users in a real-world setting.

A retrospective cohort study of 35,921 participants with overweight and obesity who used a mobile app for weight loss, which included daily meal logging, activity monitoring, and personalized feedback, found that 78% (27983/35921) of participants lost weight over a median of 8.8 months [14]. While this study had a smaller sample size, a comparable percentage of participants lost weight over a shorter time frame. One additional benefit of this study compared with the study by Chin et al [14] is the inclusion of self-reported comorbidities and program goals, which allows for further understanding of how a weight loss program impacts patients based on underlying comorbidities and program goals.

Similarly, among 8977 adults with obesity, a digital nutrition platform with personalized recipes and grocery tools resulted in a mean weight loss of 3.7 (1.5%; SD 17.4) pounds over 10 months [13]. In one-fifth the follow-up time, participants engaged with the current MNT program with a companion mobile app achieved greater mean weight loss and mean percent weight loss. Furthermore, this study includes detailed engagement data, whereas the previous study did not report engagement metrics; as a result, it remains unclear whether weight loss in the previous study was associated with active app use or passive engagement with the platform. The greater weight loss achieved in a shorter amount of time with this solution may be attributed to the integration of individualized MNT provided by RDs with a companion app. These combined modalities may provide synergistic benefits compared with stand-alone app or MNT interventions.

Recognizing the importance of a multifaceted approach to weight loss and patients’ desires to have a mobile app or web-based platform to engage with during their weight loss journey, this study aimed to better understand how app engagement further impacts weight loss success associated with MNT. The features of the mobile app component of this MNT program were designed to drive engagement and facilitate consistent behavior change to ultimately result in better adherence to diet recommendations and greater weight loss. This has been validated in the analysis by Chin et al [14], where meal logging (odds ratio [OR] 10.69, 95% CI 6.20-19.53; *P*<.001) and weight logging (OR 0.59, 95% CI 0.39-0.89; *P*<.001) were both associated with a greater likelihood of successful weight loss.

Alternatively, the trial evaluated the impact of a single 90-minute visit with an RD combined with an app for weight loss among individuals with overweight and obesity. The study concluded that the RD visit, along with app features such as meal logging and physical activity tracking via a Fitbit (Google) device, led to meaningful weight loss. However, they reported no additional benefit of receiving personalized, real-time feedback through in-app messages [24], which is in contrast to another study highlighting the benefit of an RD chat feature [25]. Therefore, while the utility of each individual app-based feature requires additional investigation, multiple studies, including this analysis, highlight that patients who additionally engaged with an app achieved the greatest weight loss.

### Limitations

There are several limitations of this study to consider. First, this study is observational and does not include a control group, which limits any causal inferences of the intervention on reported outcomes. However, this study provides valuable insights into the practical implementation and applicability of this solution across a nationwide patient population.

Second, although RDs were instructed to validate weights during each appointment, this was not a requirement and further exacerbated the limitation of self-reported data. Self-reported weights are subject to recall bias; however, previous research has suggested a moderate to strong correlation between self-reported weight data collected and actual values [26].

Third, the follow-up time was relatively short compared with other retrospective cohort studies. While longer follow-up is imperative to demonstrate the long-term utility of this program and its effect on weight loss, meaningful reductions in weight coupled with high patient engagement over several months in the program highlight the potential of this solution over an extended period.

Fourth, physical activity, which is a significant factor in achieving and maintaining weight loss, was not systematically assessed in this analysis, which prevents appropriately accounting for its impact on weight loss outcomes.

Fifth, although the program covered a diverse group of patients across the 50 states, the lack of comprehensive demographic data capture beyond age and sex limits the generalizability of these findings and prevents evaluation of the program’s effectiveness in those individuals disproportionately affected by social determinants of health.

Finally, the incomplete data capture of these variables noted above restricted adjustment for potential cofounders, thereby limiting the feasibility and interpretability of regression-based analyses.

### Conclusions

The obesity epidemic in the United States highlights the need for effective and comprehensive approaches to weight management. This study provides evidence that engagement with an MNT program and a companion mobile app is associated with clinically meaningful weight loss among individuals with overweight and obesity. It also demonstrates that combining MNT with a companion mobile app may amplify the program’s effect on weight loss. These findings highlight the potential for MNT programs with mobile apps to bridge the gap in access to effective nutrition care, offering a scalable and effective solution for weight management in individuals with overweight and obesity across the United States.

## Appendix

Multimedia Appendix 1 Percent of patients who achieved any and at least 3%, 5%, 7%, and 10% weight loss.

## Abbreviations

AHA: American Heart Association
MNT: medical nutrition therapy
OR: odds ratio
RD: registered dietitian

## Data Availability

The data analyzed in this study are proprietary and cannot be publicly shared.
