# Supplementary material for: The Impact of Technology-Enabled Medical Nutrition Therapy on Weight Loss in Adults With Overweight and Obesity: Retrospective Observational Study
Source: JMIR Mhealth Uhealth. 2025 May 6;13:e70228. doi: 10.2196/70228 (PMC12093070; doi:10.2196/70228)
Supplement: Multimedia Appendix 1 [file mhealth_v13i1e70228_app1.docx]

**Table S1.** Percent of patients who achieved any and at least 3%, 5%, 7%, and 10% weight loss.

|  | Total population (N=3,951) | <5 appointments (n=2,331) | ≥5 appointments (n=1,620) | *P, 95% CI* |
| --- | --- | --- | --- | --- |
| Achieved any weight loss, n (%) | 2,929 (74%) | 1,741 (75%) | 1,188 (73%) | 0.3, 0.0 to 4.0 |
| Achieved ≥3% weight loss, n (%) | 1,350 (34%) | 737 (32%) | 613 (38%) | <.001, 3.6 to 8.4 |
| Achieved ≥5% weight loss, n (%) | 689 (17%) | 358 (15%) | 331 (20%) | <.001, 2.8 to 7.2 |
| Achieved ≥7% weight loss, n (%) | 336 (9%) | 157 (7%) | 179 (11%) | <.001, 2.1 to 5.9 |
| Achieved ≥10% weight loss, n (%) | 123 (3%) | 50 (2%) | 73 (5%) | <.001, 1.4 to 4.6 |
